# A Negative Feedback Loop Regulates Integrin Inactivation and Promotes Neutrophil Recruitment to Inflammatory Sites

**DOI:** 10.4049/jimmunol.1900443

**Published:** 2019-08-19

**Authors:** Barry McCormick, Helen E. Craig, Julia Y. Chu, Leo M. Carlin, Marta Canel, Florian Wollweber, Matilda Toivakka, Melina Michael, Anne L. Astier, Laura Norton, Johanna Lilja, Jennifer M. Felton, Takehiko Sasaki, Johanna Ivaska, Ingeborg Hers, Ian Dransfield, Adriano G. Rossi, Sonja Vermeren

**Affiliations:** * Centre for Inflammation Research, University of Edinburgh, Edinburgh EH16 4TJ, United Kingdom;; † Babraham Institute, Babraham Research Campus, Cambridge CB22 3AT, United Kingdom;; ‡ Cancer Research UK Beatson Institute, Glasgow G61 1BD, United Kingdom;; § Institute of Cancer Sciences, University of Glasgow, Glasgow G61 1BD, United Kingdom;; ¶ Centre de Physiopathologie Toulouse-Purpan, INSERM U1043, CNRS U5282, Université Toulouse, 31024 Toulouse Cedex 3, France;; ‖ Turku Centre for Biotechnology, University of Turku, FI-20520 Turku, Finland;; # Department of Biochemical Pathophysiology, Medical Research Institute, Tokyo Medical and Dental University, Tokyo 113–8510, Japan; and; ** School of Physiology, Pharmacology and Neuroscience, University of Bristol, Bristol BS8 1TD, United Kingdom

## Abstract

Neutrophils are abundant circulating leukocytes that are rapidly recruited to sites of inflammation in an integrin-dependent fashion. Contrasting with the well-characterized regulation of integrin activation, mechanisms regulating integrin inactivation remain largely obscure. Using mouse neutrophils, we demonstrate in this study that the GTPase activating protein ARAP3 is a critical regulator of integrin inactivation; experiments with Chinese hamster ovary cells indicate that this is not restricted to neutrophils. Specifically, ARAP3 acts in a negative feedback loop downstream of PI3K to regulate integrin inactivation. Integrin ligand binding drives the activation of PI3K and of its effectors, including ARAP3, by outside-in signaling. ARAP3, in turn, promotes localized integrin inactivation by negative inside-out signaling. This negative feedback loop reduces integrin-mediated PI3K activity, with ARAP3 effectively switching off its own activator, while promoting turnover of substrate adhesions. In vitro, ARAP3-deficient neutrophils display defective PIP3 polarization, adhesion turnover, and transendothelial migration. In vivo, ARAP3-deficient neutrophils are characterized by a neutrophil-autonomous recruitment defect to sites of inflammation.

## Key Points

- A negative feedback loop, integrin–PI3K–ARAP3–integrin, controls integrin inactivation.
- Integrin inactivation promotes neutrophil transendothelial migration and recruitment.

## Introduction

Neutrophils are abundant leukocytes that are key to the inflammatory response and provide a first line of defense against infections. Upon stimulation, circulating neutrophils leave the blood stream to be recruited to sites of infection or injury, where they phagocytose and kill pathogens, releasing reactive oxygen species (ROS) and other cytotoxic agents (1, 2). Inappropriately activated neutrophils can make important contributions to host injury.

Integrins are α/β heterodimeric cell surface receptors that bind to extracellular matrix proteins and transmembrane receptors expressed by activated endothelial cells, bridging them to the cytoskeleton (3). In addition to the major β2 leukocyte integrins, neutrophils also express others, including the ubiquitous β1 integrins. Integrin ligation triggers “outside-in” signaling to initiate intracellular signaling cascades. This is distinct from “inside-out” signaling, which refers to intracellular signaling events that regulate the integrin ligand binding affinity status. Although the mechanism of integrin activation is well characterized in leukocytes, the regulation of integrin inactivation remains largely elusive.

Integrins are essential for neutrophil recruitment to sites and clearance of infections, as illustrated by leukocyte adhesion deficiencies, rare genetic diseases characterized by lacking, dysfunctional, or activation-impaired β2 integrins (4, 5). A large body of work identified how leukocyte integrins are activated in a mechanism that is crucial for neutrophil recruitment to inflamed sites. Proximally, this involves the adapters talin and kindlin-3, which directly bind to integrin cytoplasmic tails, promoting their activation (6, 7), with Rap and its effectors, RAPL, RIAM, and Radil, acting upstream. Excessive integrin activity has also been shown to interfere with leukocyte recruitment (8, 9), but mechanisms governing integrin inactivation in this context remain poorly defined.

Class I (agonist-activated) PI3Ks transduce signals through the generation of the lipid second messenger phosphatidylinositol 3,4,5-trisphosphate (PIP3) by phosphorylation of PI(4,5)P2 in the plasma membrane. Four class I PI3K isoforms exist and are expressed by the neutrophil: α, β, γ, and δ (10). Class I PI3K isoforms are activated upon receptor ligation by SH2 domain binding to phosphotyrosine motifs in receptors or their adapters (e.g., in integrin outside-in signaling) and G protein βγ subunits, as well as Ras/Rho family small GTPases. PIP3 causes the recruitment to the plasma membrane and activation of numerous PI3K effector proteins, including several regulators of small GTPases.

ARAP3 is a PI3K- and Rap-regulated GTPase activating protein for RhoA and Arf6 that was identified as a PIP3 binding protein from pig neutrophils (11, 12). ARAP3 shares its domain structure with ARAP1/2, which differ in their expression profiles and substrate specificities (11, 13–15). We previously showed ARAP3 to regulate adhesion-dependent processes in the neutrophil (16). The data presented in this study identify that integrin activation triggers a negative feedback loop downstream of PI3K by which ARAP3 promotes integrin inactivation. Despite focusing in this study on β1 integrins in neutrophils, we demonstrate that this function of ARAP3 is also broadly applicable elsewhere. As well as causing a polarization and chemotaxis defect in vitro, in vivo, ARAP3-deficiency interferes with efficient neutrophil recruitment to sites of inflammation.

## Materials and Methods

Unless indicated otherwise, cell culture reagents were from Life Technologies, cell culture plastics were from Corning, and all other materials were from Sigma. All reagents were of the lowest available endotoxin grade. PI3K inhibitors (Selleck Chemicals) and final concentrations used were as follows: pan-PI3K, wortmannin (50 nM); PI3Kα, BYL-719 (0.25 μM); PI3Kβ, TGX-221 (40 nM); and PI3Kδ, IC87114 (1 μM).

### Inducible *Arap3^−/−^* mouse model

To analyze neutrophils in vitro, 10–12-wk-old sex-matched *Arap3*^fl/fl^ *ERT2Cre*^+^ mice were induced with a single i.p. injection with 200 mg/kg tamoxifen or vehicle, with experiments performed 10–12 d after induction as described (16). For in vivo experiments, age- and sex-matched *Arap3*^fl/fl^ *ERT2Cre*^+^ mice and *Arap3*^+/+^*ERT2Cre*^+^ controls were subjected to five successive gavages with emulsion containing 1.5 mg of tamoxifen, followed by a rest period of 10 d (Supplemental Fig. 3A for an example). For ease of reading, tamoxifen-induced *Arap3*^fl/fl^ *ERT2Cre*^+^ mice (or neutrophils) are referred to in the text as ARAP3-deficient and in figures as ^−/−^, whereas vehicle-induced *Arap3*^fl/fl^ *ERT2Cre*^+^ and tamoxifen-induced *Arap3*^+/+^*ERT2Cre*^+^ controls are referred to as controls and ^+/+^, with explanations provided in the figure legends. All mice were housed in a specific pathogen–free small animal barrier unit at the University of Edinburgh. All animal work was approved by the University of Edinburgh Animal Welfare Committee and conducted under the control of the U.K. Home Office (PPL 60/4502 and PFFB 42579).

### Neutrophil preparations

Bone marrow–derived mouse neutrophils were prepared on a discontinuous Percoll gradient as previously described (17), using endotoxin reagents throughout, yielding ∼70% purity as assessed by cytocentrifuge preparations. Unless stated otherwise, experiments were performed in Dulbecco’s PBS supplemented with Ca^2+^ and Mg^2+^, 1 g/l glucose, and 4 mM sodium bicarbonate.

### Adhesion-induced neutrophil functions

Tissue culture wells were coated overnight at 4°C with fibronectin as indicated. Surfaces were washed three times with PBS, blocked with 10% FBS in PBS, and washed again before addition of prewarmed neutrophils. ROS production was measured indirectly using chemiluminescence produced by 5 × 10^5^ neutrophils per well at 37°C with 150 μM luminol and 18.75 U/ml HRP in the presence or absence of TNF-α (20 ng/ml final concentration) in luminescence-grade 96-well plates (Nunc) using a Cytation plate reader (BioTek) essentially as described (16). Where indicated, neutrophils were preincubated with inhibitors for 10 min at 37°C at the indicated concentrations. Where blocking peptides were employed, neutrophils were plated onto the immobilized stimuli and the competing peptide, such that both were encountered at the same time. Neutrophil adhesion, spreading, and degranulation assays were done as previously described (16). For adhesion to endothelial cells, bEND5 cells were seeded into 24 wells, allowed to form confluent monolayers for 2 d, and stimulated with 5 nM TNF-α for 16 h. After washing and careful aspiration, 100 μl of HBSS (with Ca^2+^ and Mg^2+^) containing 1 × 10^5^ neutrophils were added and allowed to bind to the stimulated endothelial cells under gentle rocking. After 30 min, nonadherent neutrophils were washed away with HBSS (without Ca^2+^ and Mg^2+^). Adherent neutrophils were fixed with PFA, labeled for GR1 (clone RB6-8C5; BioLegend), and counted in randomly taken frames (EVOS imaging system; Advanced Microscopy Group/Thermo Fisher). Transendothelial migration toward the indicated concentrations of MIP2 (R&D Systems) for 1 h in 6.5-mm transwell inserts with 3-μm pore polycarbonate membranes (Corning) was performed as described (17). Transmigrated neutrophils were labeled for GR1, and eight random fields of view were photographed and counted (×20 magnification; EVOS imaging system).

### ARAP3 knockdown in αIIbβ3-expressing Chinese hamster ovary cells

αIIbβ3-Expressing Chinese hamster ovary (CHO) cells were transduced with lentiviral short hairpin RNAs (shRNAs) directed against mouse ARAP3. shRNA sequences (shRNA1, 5′-CTCCGGCTGGAAGGTGTATAT-3′ and 5′-GGAATCCGCAAGAAGTTAAA-3′; shRNA2, 5′-GCAGAAGTGTGCGTCTCTAAA-3′ and 5′-TGTATGAAGAGCCAGTATATG-3′) identified from the Broad Institute RNA interference consortium database (https://portals.broadinstitute.org/gpp/public) were used alongside a nontargeting control (NTC; 5′-GCGCGATAGCGCTAATAATTT-3′). Oligonucleotides were synthesized (Sigma-Genosys) and cloned into pLKO.1 (18), inserts were sequenced, lentiviral particles were generated, and transduced CHO cell populations were selected with puromycin. Samples were analyzed by Western blot using sheep anti-ARAP3 antiserum (11) and anti-human CD41 (MAB7616; R&D Systems), with HSP90 (clone 3H3C27; BioLegend) serving as loading control.

### CHO cell adhesion and spreading

Trypsinized CHO cells in Dulbecco’s PBS supplemented with Ca^2+^ and Mg^2+^, 1 g/l glucose, and 4 mM sodium bicarbonate were preincubated with inhibitors or vehicle for 10 min at 37°C as indicated prior to being plated for 30 min onto glass coverslips that had been coated with 150 μg/ml fibrinogen and blocked with 2% fatty acid–free BSA. Fixed, washed cells were stained with AF568-conjugated phalloidin (Thermo Fisher Scientific); random images were acquired at ×20 magnification (EVOS imaging system). Prior to measuring cell areas with ImageJ, binary images were thresholded, and the watershed feature was applied to define single cells.

### Direct analysis of integrin activity status

Activated β1 integrin was detected using an activation epitope–specific Ab (clone 9EG7; BD Biosciences) with an AF488-conjugated secondary Ab (Invitrogen). Images were acquired with a 63× objective using a Zeiss LSM780 confocal microscope with Zeiss Zen Black software. The corrected total cellular fluorescence was calculated using ImageJ by selecting regions for each cell and nearby regions of background and applying the following formula: corrected total cellular fluorescence = integrated density – (area of selected cell × mean fluorescence of background readings).

Neutrophil binding to an AF647-labeled fibronectin fragment was performed essentially as described (19) using flow cytometry using a 5L LSRFortessa (BD Biosciences). Analysis was performed using FlowJo software (version 10) by gating for singlets, selecting neutrophils based on forward- and side-scatter profile, and measuring the geometric mean fluorescence intensity. Similarly, CHO cell binding to AF594-labeled fibrinogen (Thermo Fisher Scientific) as well as activated and total αIIbβ3 on trypsinized CHO cells were detected with fluorescently conjugated Abs (clones PAC-1 and A2A9.6, respectively; BioLegend) and analyzed by measuring the geometric mean fluorescence intensity of singlets.

### Indirect analysis of PI3K activity

Neutrophil lysates were subjected to immunoblotting with a phosphospecific anti-PKB T308 (clone C25E6; Cell Signaling Technology) essentially as described (17), with Syk used as loading control (clone 5F5; BioLegend).

### Analysis of GFP–PH–PKB reporter distribution

Micropipette chemotaxis assays were conducted, and polar plots were derived and overlaid using Anagraph (S. Andrews, The Babraham Institute) and QuimP software (20) (Garching Innovation) as described (21).

### Neutrophil adhesion under laminar flow conditions

Purified neutrophils were preincubated for 10 min at 37°C with PI3K inhibitors or vehicle as indicated prior to being perfused through flow chamber slides (Ibidi VI^0.4^) that had been coated with recombinant murine (rm) ICAM-1 (15 μg/ml), rm P-selectin (10 μg/ml; both BioLegend), and rm CXCL1 (10 μg/ml; Biotechne) using a syringe pump (Legato 200; KD Scientific) to deliver a constant shear stress of 1 dyne/cm^2^ at 37°C. Adhesion under flow was recorded with ×20 magnification by time lapse imaging (2.5 images/s) for 1 min at 1, 5, 10, and 15 min after starting the flow. This was done using a Leica IRB inverted microscope equipped with a temperature-controlled automated stage (Prior), an Orca camera (Hamamatsu), and Micro-Manager image acquisition software (Fiji). Firmly adherent cells were manually counted using ImageJ.

### LPS-induced acute lung inflammation

LPS-induced acute lung inflammation (ALI) was performed essentially as described (22). Some mice received 3 μg of allophycocyanin–anti-CD45 (30-F11; BioLegend) in 100 μl of sterile PBS i.v. 15 min prior to being sacrificed 4 h after LPS administration, such that in lung digest samples, neutrophils could be stratified by CD45^+^ and CD45^−^ staining, indicating vascular or interstitial cells, respectively. Lungs were slowly perfused through the right ventricle with 10 ml of saline, and a portion of the right inferior lobe was collected for single-cell digestion with collagenase (Roche) and subsequent analysis. Bronchoalveolar lavage (BAL) cells were counted (NucleoCounter; Sartorius). BAL cells and lung digests were labeled with FITC–anti-GR1 and allophycocyanin–anti-CD11b (BioLegend) and analyzed by flow cytometry to calculate total neutrophil numbers (GR1^high^, CD11b^+^). For imaging, lungs were perfused with low-melting-point agarose, allowed to set on ice, dissected, and fixed with formaldehyde. Left lungs were precision-sliced 300 μm thick using a vibratome (5100 MZ; Campden Instruments), permeabilized, blocked, and labeled for PECAM-1 (clone 2H8; Abcam) and S100A9/MRP14 (Hycult Biotech) with DAPI counterstaining. Following brief formaldehyde postfixation, slices were mounted using Mowiol containing 2.5% (w/v) DABCO in gaskets and analyzed using a confocal laser scanning microscope to produce tile-scanned *z*-stacks (LSM 880 nonlinear optical Airyscan Fast using a 20× plan Apo 1.0 numerical aperture, water immersion objective and 405-, 488-, and 561-nm continuous wave lasers and acquiring in Airyscan Fast mode; Zeiss). Image analysis was performed using IMARIS software (versions 8 and 9, Bitplane; Oxford Instruments). Endothelial surfaces (PECAM-1^+^) were rendered to allow identification of airway, interstitital, or vascular compartments. Vascular and perivascular neutrophils (S100A9^+^) were counted and normalized to the total volume of the vasculature.

### Statistical analysis

Where data met the assumptions for parametric tests, two-tailed Student *t* tests or one-way ANOVA with Bonferroni-corrected post hoc comparisons was used. Otherwise, the nonparametric Mann–Whitney rank sum test was used for comparisons. For multiple comparisons, ANOVA with Bonferroni-corrected post hoc comparisons was used. For kinetic experiments (ROS production), the area under the curve was calculated, excluding baseline measures, and comparisons were made using a two-tailed Student *t* test. The *p* values <0.05 were considered statistically significant.

## Results

We previously described an embryonically lethal *Arap3*-knockout mouse (23) and a tamoxifen-inducible system for the analysis of ARAP3-deficient neutrophils. Apart from leukocyte-specific β2 integrins, neutrophils express many others, including ubiquitous β1 integrins that are involved in interactions with extracellular matrix components such as fibronectin and vitronectin. In keeping with our earlier work, we observed enhanced effector functions, including adhesion, spreading, ROS production, and degranulation, with ARAP3-deficient neutrophils that had been plated onto fibronectin with costimulation by TNF-α (Supplemental Fig. 1A–G) but not upon stimulation with formylated peptides (16). This implies that ARAP3 is an important regulator of neutrophil functions downstream of β1 integrin ligation.

### ARAP3 promotes neutrophil β1 integrin inactivation

**FIGURE 1.**
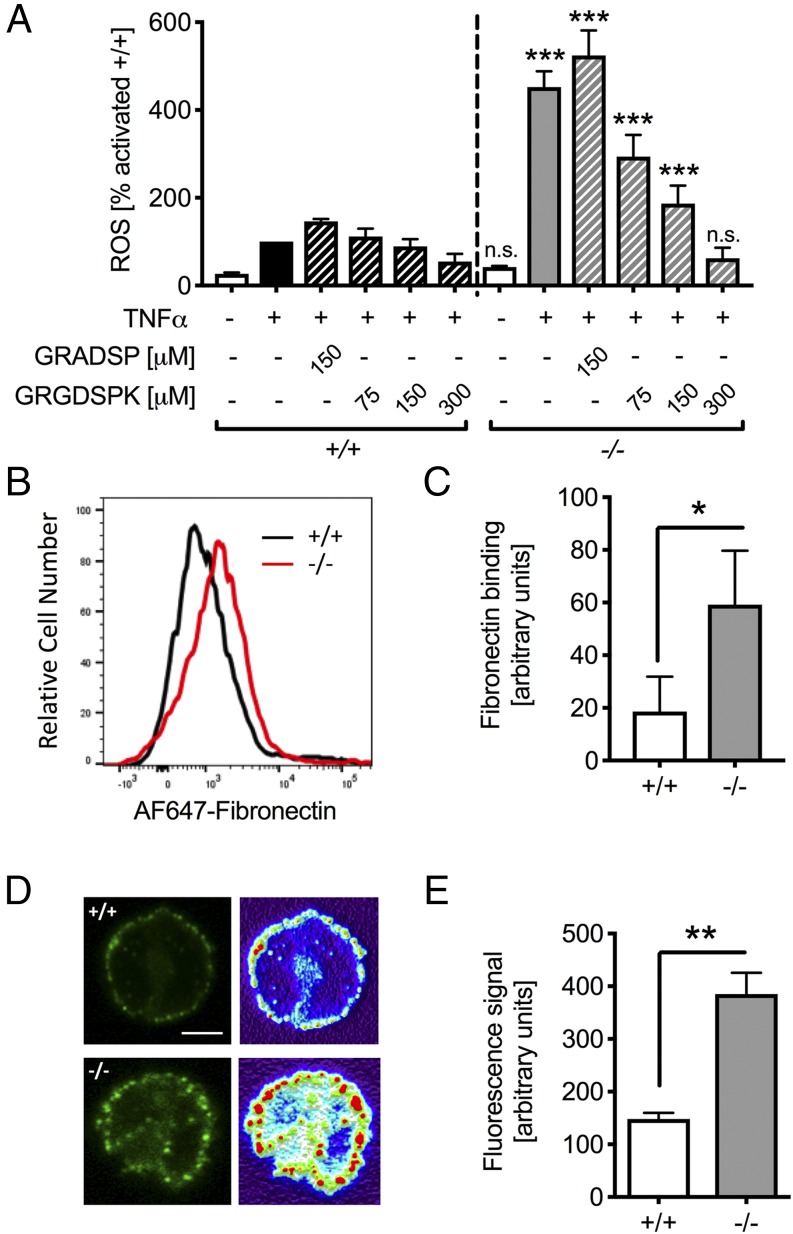
ARAP3 promotes β1 integrin inactivation in neutrophils. Neutrophils were prepared from bone marrow of mock (^+/+^) and tamoxifen-induced (^−/−^) inducible *Arap3*-knockout mice. (**A**) ROS production was analyzed with neutrophils that had been plated onto 20 μg/ml fibronectin in the presence or absence of 20 ng/ml TNF-α together with the indicated concentration of the RGD blocking peptide GRGDSPK or the control peptide GRADSP. Results obtained in four separate experiments are combined in this graph. (**B** and **C**) Binding of control and ARAP3-deficient neutrophils to a fluorochrome-coupled fibronectin fragment was determined by flow cytometry. A representative experiment (B) and the integrated results from four separate experiments (C) are presented. (**D** and **E**) Neutrophils were allowed to adhere to fibronectin-coated coverslips, fixed, and immunostained with a β1 activation epitope–specific Ab. Representative confocal images with corresponding heatmaps of the fluorescence intensity are shown (D). Scale bar, 5 μm. (E) Integrated results obtained with 9–18 cells analyzed per genotype from three separately performed experiment are plotted. All bar graphs show mean ± SEM. *p < 0.05, **p < 0.01, ***p < 0.001, calculated by unpaired two-tailed Student *t* tests.

To ascertain whether the hyper-stimulatory effect of fibronectin binding on ARAP3-deficient neutrophils was due to integrin activity, we made use of a blocking peptide, GRGDSPK, that has been shown to compete with fibronectin binding (24, 25). GRGDSPK, but not a control peptide with disrupted arginine/glycine/aspartic acid (RGD) motif, interfered with ROS production induced by plating control and ARAP3-deficient neutrophils onto fibronectin-coated plastic in the presence of TNF-α in a concentration-dependent fashion (Fig. 1A).

Increased integrin abundance might explain such increased responses. We did, however, not observe any increased surface integrin expression with ARAP3-deficient neutrophils [Supplemental Fig. 1H, 1J, data not shown (16)]. An alternative explanation would be an activated integrin conformation present in ARAP3-deficient neutrophils. We analyzed binding of suspension control and ARAP3-deficient neutrophils to a fluorescently tagged soluble fibronectin fragment. In the presence of 1 mM Mg^++^, ARAP3-deficient neutrophils exhibited a significant increase in fibronectin fragment binding compared with controls (Fig. 1B, 1C). We also employed an Ab that binds to an activation epitope present on both human and mouse β1, 9EG7. We plated control and ARAP3-deficient neutrophils onto fibronectin in the presence of TNF-α and observed significantly increased 9EG7 binding with ARAP3 deficiency (Fig. 1D, 1E). We concluded that ARAP3 promotes integrin inactivation in the neutrophil.

### ARAP3 promotes inactivation of heterologous human αIIbβ3 and endogenous integrins in CHO cells

**FIGURE 2.**
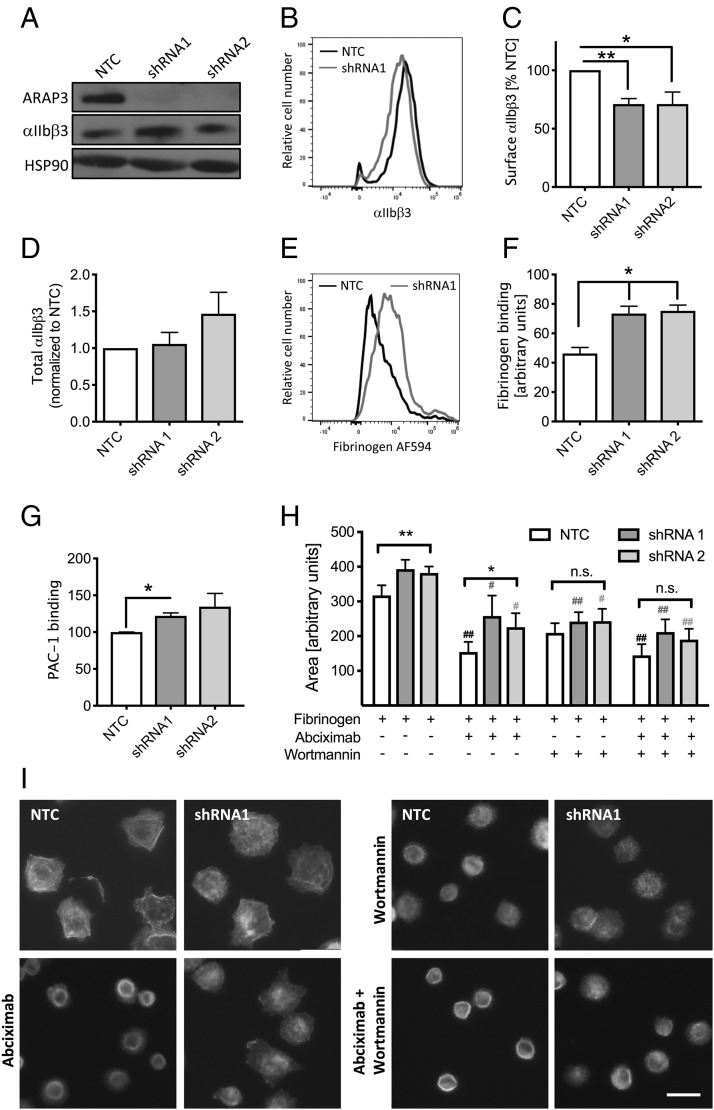
ARAP3 promotes inactivation of heterologous human αIIbβ3 integrin in CHO cells. (**A**) CHO cells were transduced to express two distinct pools of shRNAs directed against mouse ARAP3 or an NTC. A representative Western blot is shown; HSP90 served as a loading control. (**B** and **C**) Surface αIIbβ3 on CHO cell populations was determined by flow cytometry. A representative example (B) and integrated results from three separately performed experiments are plotted (C). (**D**) Integrated results from three separately performed Western blots for total cellular αIIbβ3 expression. (**E** and **F**) CHO cell binding to fluorescently tagged fibrinogen was analyzed in suspension cultures. A representative example is shown (E), together with results integrated from at least five separately performed experiments (F). (**G**) Activation epitope–specific PAC1 staining normalized to the total cell surface αIIbβ3 in each cell population. Integrated results from three separately performed experiments are presented. (**H** and **I**) Control and ARAP3-knockdown αIIbβ3-expressing CHO cells that had or had not been preincubated with the αIIbβ3-blocking Ab abciximab or the pan-PI3K inhibitor wortmannin, as indicated, were allowed to adhere to fibrinogen-coated coverslips. Random images were taken, and the cell areas were analyzed. Results from four to seven separate experiments are plotted (H), together with representative images (I). Scale bar, 20 μm. All bar graphs show mean ± SEM. Raw data were analyzed for statistical significance. The *p* values were calculated by one-way ANOVA with Bonferroni-corrected post hoc testing (C, D, F, and G), and data were analyzed by two-way ANOVA with Bonferroni posthoc test, respectively (H). Significant differences between treatments of the same populations are indicated above the individual bars with hashtag symbols, whereas differences between NTC and shRNA-expressing cells within each condition are indicated by asterisk symbols above the brackets. ^#,^*p < 0.05, ^##,^**p < 0.01.

ARAP3 expression is restricted to some myeloid cells and the vasculature in the mouse (data not shown), but it is more broadly expressed in epithelial cells in some other organisms (11). To establish whether ARAP3-mediated integrin inactivation is restricted to the neutrophil, we used CHO cells that had been engineered to express the human platelet integrin αIIbβ3 (26). Taking advantage of the high degree of conservation between hamster and mouse ARAP3 (92% cDNA identity), we generated two ARAP3-knockdown CHO cell populations by expressing distinct pools of mouse ARAP3-targeting shRNAs alongside a population expressing an NTC shRNA (Fig. 2A).

Surface (but not total) human αIIbβ3 was reduced in both ARAP3 knockdowns (Fig. 2A–D). To test whether ARAP3 regulates the activity status of the heterologous αIIbβ3 in CHO cells, we measured binding to fluorescently labeled fibrinogen by suspending CHO cells by flow cytometry. Increased fibrinogen binding was observed with both ARAP3-knockdown populations (Fig. 2E, 2F). Moreover, by employing the activation epitope–specific Ab PAC-1, we noted that the proportion of activated out of total surface αIIbβ3 was increased in ARAP3-knockdown cells (Fig. 2G), consistent with the notion that ARAP3 regulates inactivation of heterologous human αIIbβ3 integrin in CHO cells, too.

In cancer cells, increased β1 integrin activity correlates with increased spreading (19). As an indirect readout of integrin activity, we therefore also measured the areas occupied by CHO cells that had been plated onto fibrinogen (Fig. 2H, 2I). ARAP3-knockdown CHO cells occupied a significantly larger area than NTC-expressing CHO cells, again indicative of ARAP3-dependent control of CHO cell integrins. Preincubating the cells with αIIbβ3-blocking abciximab significantly reduced the area occupied by control and ARAP3-knockdown CHO cells, suggesting that heterologous αIIbβ3 mediated most fibrinogen binding. Interestingly, however, abciximab-preincubated ARAP3-knockdown cells remained more spread than controls, suggesting that ARAP3 inactivates not only αIIbβ3 but also endogenous hamster integrins that were also capable of binding fibrinogen without being affected by the blocking Ab. Inhibiting PI3K significantly reduced the areas occupied by control and ARAP3-knockdown CHO cells. No significant difference remained between experimental groups after treatment with wortmannin. These observations are in keeping with ARAP3 being a PI3K effector that is able to regulate many integrins, including heterologous human αIIbβ3 in CHO cells.

### ARAP3 acts in a negative feedback loop downstream of integrin and PI3K

Having established that ARAP3 mediates integrin inactivation, we turned our attention to upstream signaling. In the neutrophil, ARAP3’s master regulator, PI3K, is activated by integrin outside-in signaling downstream of Src family kinases/Syk (27), with PI3Kβ and δ isoforms implicated in mediating integrin-dependent responses (28).

**FIGURE 3.**
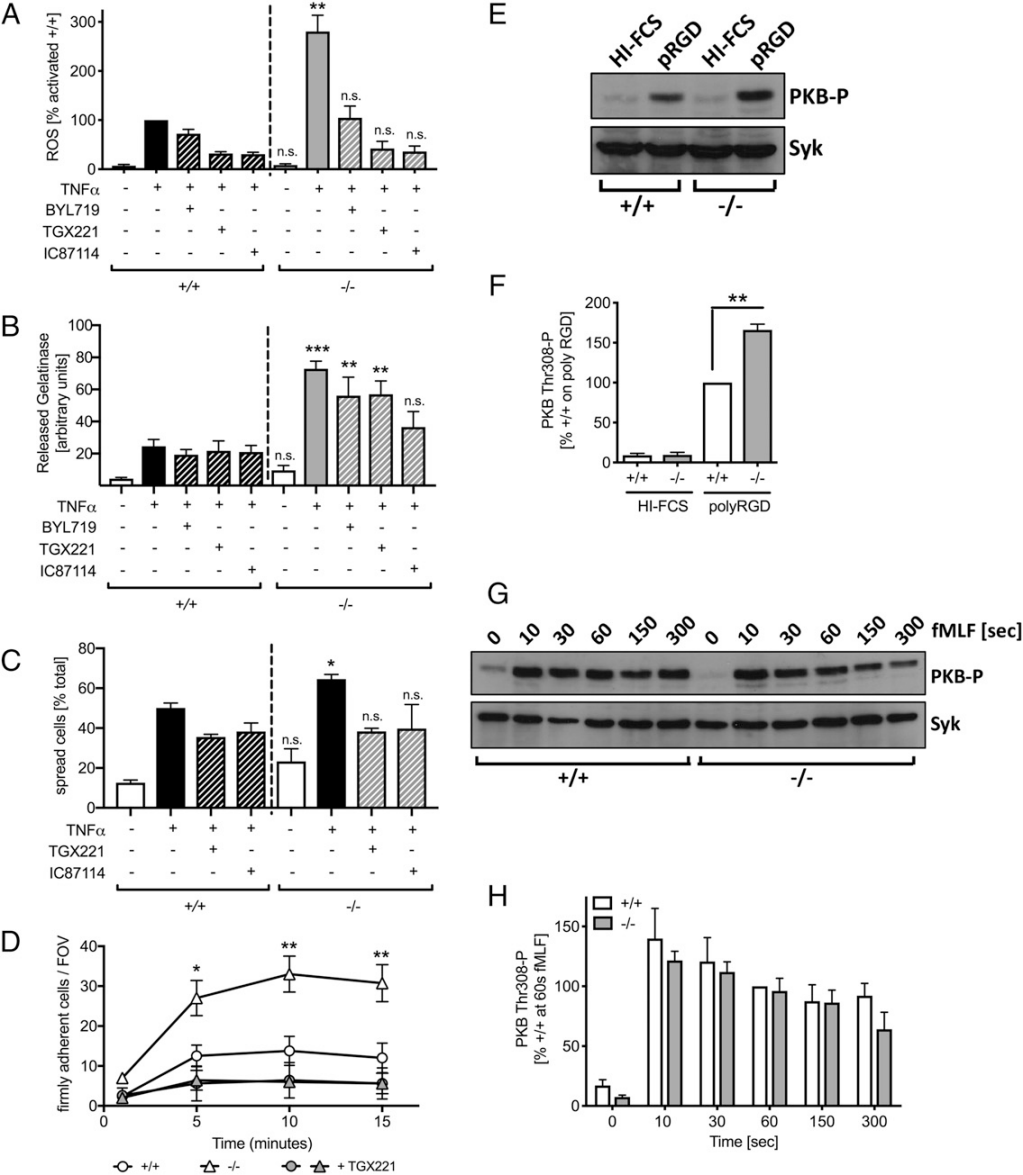
A negative feedback loop involving integrin, PI3K, and ARAP3. Neutrophils were prepared from bone marrow of mock (^+/+^) and tamoxifen-induced (^−/−^) inducible *Arap3*-knockout mice and (**A**–**D**) preincubated with PI3K inhibitors or vehicle controls as indicated. (A) ROS production and (B) gelatinase release were analyzed with neutrophils that had been plated onto 20 μg/ml fibronectin in the presence or absence of 20 ng/ml TNF-α. Graphs combine results from four separate experiments. (C) Neutrophils were allowed to adhere for 20 min to 5 μg/ml fibronectin-coated tissue culture plastic in the presence or absence of 20 ng/ml TNF-α for analysis of spreading. Results obtained in three separate experiments are integrated in this graph. (D) Neutrophil adhesion under flow. Neutrophils were perfused at constant shear stress through ICAM-1–, P-selectin–, and CXCL1-coated flow chambers as detailed in *Materials and Methods*. Results obtained in at least five separate experiments are combined in the graph shown. (**E** and **F**) Neutrophils were allowed to adhere to tissue culture dishes that had been coated with heat-inactivated FCS (HI-FCS) or a synthetic pan-integrin ligand, poly-RGD (pRGD) for 15 min at 37°C. (**G** and **H**) Suspension neutrophils were stimulated with 1 μM fMLF for the indicated length of time. Lysates were subjected to SDS-PAGE and Western blots for probing with a phosphospecific Akt/PKB Ab (T308) as well as a loading control (Syk). Representative blots are shown (D and F), and results obtained from four separately performed experiments are plotted (E and G). All graphs show mean ± SEM. (A)–(C) were analyzed by one-way ANOVA with multiple-comparison post hoc tests; (D) and (H) were analyzed by two-way ANOVA with Bonferroni multiple-comparison tests. Pairwise comparisons (F) were calculated from raw data by unpaired two-tailed Student *t* tests. (A, F, and H) Analyses were performed on the raw data. Symbols in graphs (A)–(D) refer to differences between control and ARAP3-deficient neutrophils (in the absence of inhibitor treatment). No significant differences between genotypes were identified in (H). *p < 0.05, **p < 0.01, ***p < 0.001.

To probe the relationship between integrin, PI3K, and ARAP3, we analyzed ROS production with neutrophils that had been plated onto fibronectin in the presence or absence of TNF-α. Integrin ligation-induced ROS depends on PIP3 generation through class I PI3K, in particular PI3Kβ and δ (28), whereas SHIP1 (29) or ARAP3 (Fig. 1) deficiency causes increased adhesion-dependent ROS. Inhibiting individual class IA PI3K isoforms reduced adhesion-induced ROS production observed with control and ARAP3-deficient neutrophils and abrogated significant differences observed between genotypes (Fig. 3A).

ROS production is dependent on PIP3-activated Rac guanine nucleotide exchange factors, inhibition of which could potentially explain the above result. We therefore also analyzed the PI3K dependency of degranulation with control and ARAP3-deficient neutrophils that been stimulated by being plated onto fibronectin in the presence or absence of TNF-α. Inhibiting class IA PI3Ks also reduced the enhanced degranulation that is characteristic of ARAP3-deficient cells; in particular, following PI3Kδ inhibition, no significant difference remained between genotypes (Fig. 3B).

We analyzed adhesion and spreading of control and ARAP3-deficient neutrophils after PI3K inhibition to fibronectin-coated plastic. Inhibiting PI3Kβ/δ did not significantly affect the ability of neutrophils to adhere to fibronectin, in keeping with an earlier report that had analyzed neutrophil adhesion to immobilized immune complexes [(28), data not shown]. However, it resulted in compromised neutrophil spreading in both genotypes, putting an end to significant differences between them (Fig. 3C).

Finally, we compared adhesion of neutrophils under constant flow in parallel-plate flow chambers. As previously reported (16), we noted increased neutrophil adhesion with ARAP3-deficient neutrophils compared with controls. Preincubating the neutrophils with a PI3Kβ-specific inhibitor caused decreased neutrophil adhesion in both genotypes (Fig. 3D). Notably, this abolished the significant difference in adhesion observed between genotypes in the absence of inhibitor treatment. Together, these results show that ARAP3 acts downstream of PI3K in neutrophil adhesion and adhesion-dependent neutrophil functions. Given the heightened responses observed with ARAP3-deficient neutrophils, they also suggest the existence of a negative feedback loop.

For experimental evidence of this feedback loop, we analyzed PKB/Akt T308 phosphorylation as an indirect readout for PI3K activity with neutrophils that did or did not express ARAP3. PKB T308 phosphorylation was increased more dramatically in ARAP3-deficient than control neutrophils that had been plated onto the synthetic integrin ligand poly-RGD (Fig. 3E, 3F). In contrast, ARAP3 deficiency did not confer increased PKB T308 phosphorylation in neutrophils that had been stimulated with the soluble agonist fMLF (Fig. 3G, 3H). We concluded that ARAP3 functions in a negative feedback loop specifically downstream of integrin-stimulated PI3K to inactivate integrins.

### Integrin–PI3K–ARAP3 negative feedback signaling regulates persistent neutrophil polarization during chemotaxis

**FIGURE 4.**
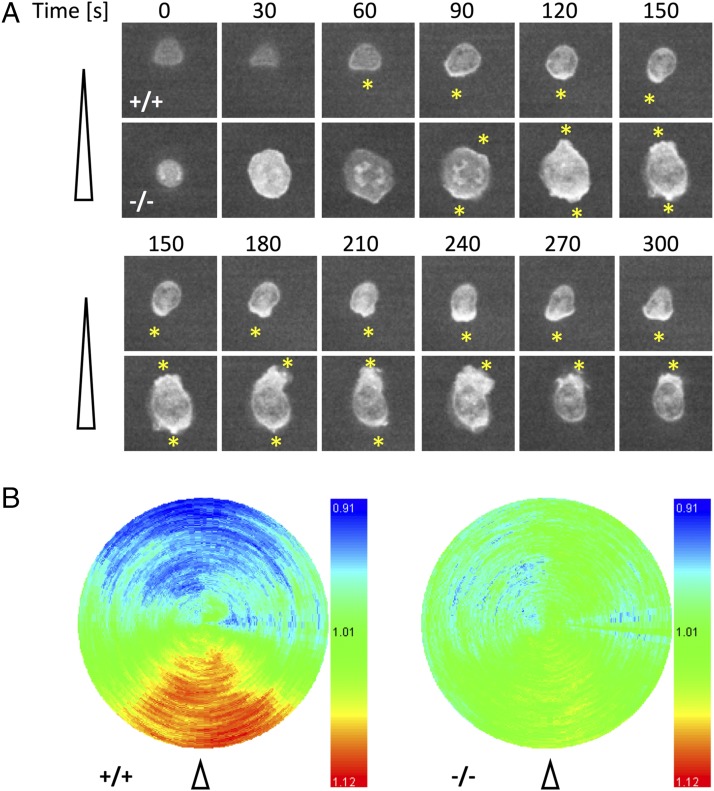
Integrin–PI3K–ARAP3 negative feedback signaling improves neutrophil polarization. Neutrophils were prepared from bone marrow of mock (^+/+^) and tamoxifen-induced (^−/−^) inducible *Arap3*-knockout mice expressing a GFP–PKB–PH PIP3 reporter. Cells were allowed to settle on a glass coverslip and then subjected to a point source of chemoattractant (micropipette). Cells were imaged using a Perkin Elmer spinning disk Nikon Eclipse TE2000E confocal microscope using a 100× oil immersion objective. Images were acquired every second for 5 min using a Hamamatsu cooled charge-coupled device camera. (**A**) Stills taken from a representative control and ARAP3-deficient neutrophil. Yellow asterisk symbols indicate polarization. (**B**) The distribution of the PIP3 probe along the edge of each frame of the video was analyzed using QuimP software, measuring the image intensity at 100 nodes around the plasma membrane. The signal intensity along the membrane was normalized to that within the cell body. Intensity measurements were plotted using Anagraph, with each frame mapped onto a concentric ring and signal intensity represented by color-coding to generate polar plots. The images shown represent overlays of polar plots generated with 25 control, and 24 ARAP3-deficient neutrophils originating from six individual animals per genotype.

Chemotaxing neutrophils are characterized by polarized PIP3 at the pseudopod (30, 31). To analyze whether the negative feedback loop delineated in this study operates to control neutrophil behavior, we analyzed PIP3 generation in the chemotaxing neutrophil in a spatiotemporal fashion. Having crossed inducible ARAP3-knockout mice with mice expressing a PIP3 probe, GFP–PKB–PH (30), we used confocal microscopy to monitor PIP3 production in real time in control and ARAP3-deficient neutrophils that were allowed to chemotax on glass coverslips toward fMLF. Control cells displayed persistent PIP3 polarization toward the chemoattractant. In contrast, ARAP3-deficient cells were unable to polarize PIP3 persistently, with poles observed to move around cells; more than 50% of ARAP3-deficient neutrophils exhibited additional poles (Fig. 4A for an example). We generated polar plots (21, 31), to visualize PIP3 polarization over time in individual neutrophils (data not shown). Overlays of these polar plots confirmed the poor persistency of PIP3 polarization of ARAP3-deficient neutrophils (Fig. 4B).

In the absence of a probe for activated integrins, we were unable to test whether nonpersistent PIP3 polarization of ARAP3-deficient neutrophils coincided with poor turnover of activated integrins. Fixed, adherent fMLF bath-stimulated control, and ARAP3-deficient neutrophils were characterized by polarized activated β1 integrin staining at the pseudopod, where it coincided with F-actin (Supplemental Fig. 2). For efficient forward motion of the neutrophil, these adhesions must be short-lived. Given that ARAP3 is recruited to the plasma membrane by PIP3 (11), it is well placed to be involved in localized integrin inactivation, ensuring persistence of polarization and directionality.

### ARAP3 regulates neutrophil transendothelial migration and recruitment to sites of inflammation

**FIGURE 5.**
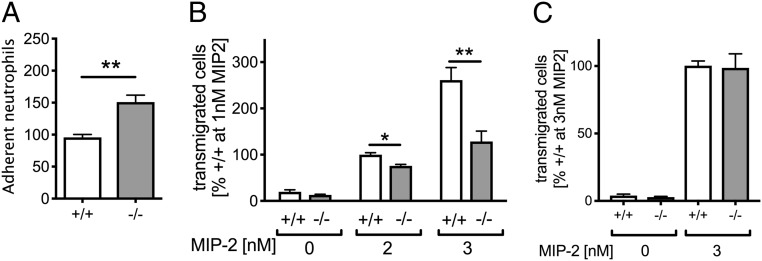
ARAP3-regulated integrin inactivation promotes transendothelial migration in vitro. Neutrophils were prepared from bone marrow of mock (^+/+^) and tamoxifen-induced (^−/−^) inducible *Arap3*-knockout mice. Neutrophil adhesion (**A**) to activated mouse endothelial (bEND5) cells. Neutrophil transendothelial migration and chemotaxis (**B** and **C**) toward the indicated concentrations of chemoattractant in transwells that did (B) or did not (C) support a monolayer of activated bEND5 cells. Graphs integrate data obtained from three to four separate experiments. All bar graphs show mean ± SEM. Pairwise comparisons were analyzed by unpaired two-tailed Student *t* tests. *p < 0.05, **p < 0.01.

We next determined the requirement for ARAP3-dependent integrin inactivation in neutrophil recruitment to inflammatory sites. Whereas interstitial migration is thought to be integrin-independent, barriers need to be overcome in an integrin-dependent fashion for neutrophil recruitment (e.g., during transendothelial migration). We first addressed whether the increased integrin activity of ARAP3-deficient neutrophils influences interactions with endothelial cells and transendothelial migration efficiency in vitro. As expected, we found that ARAP3-deficient neutrophils adhered more strongly than controls to monolayers of activated endothelial cells (Fig. 5A). Furthermore, ARAP3-deficient neutrophils were characterized by impaired migration to chemoattractant in a model for transendothelial migration, where transwells supported a monolayer of TNF-α–stimulated endothelial cells (Fig. 5B). In contrast, ARAP3-deficient neutrophils were not defective in transwell chemotaxis (Fig. 5C), in line with our previous findings. Together this suggested that ARAP3-dependent integrin inactivation might be relevant for neutrophil recruitment in vivo.

**FIGURE 6.**
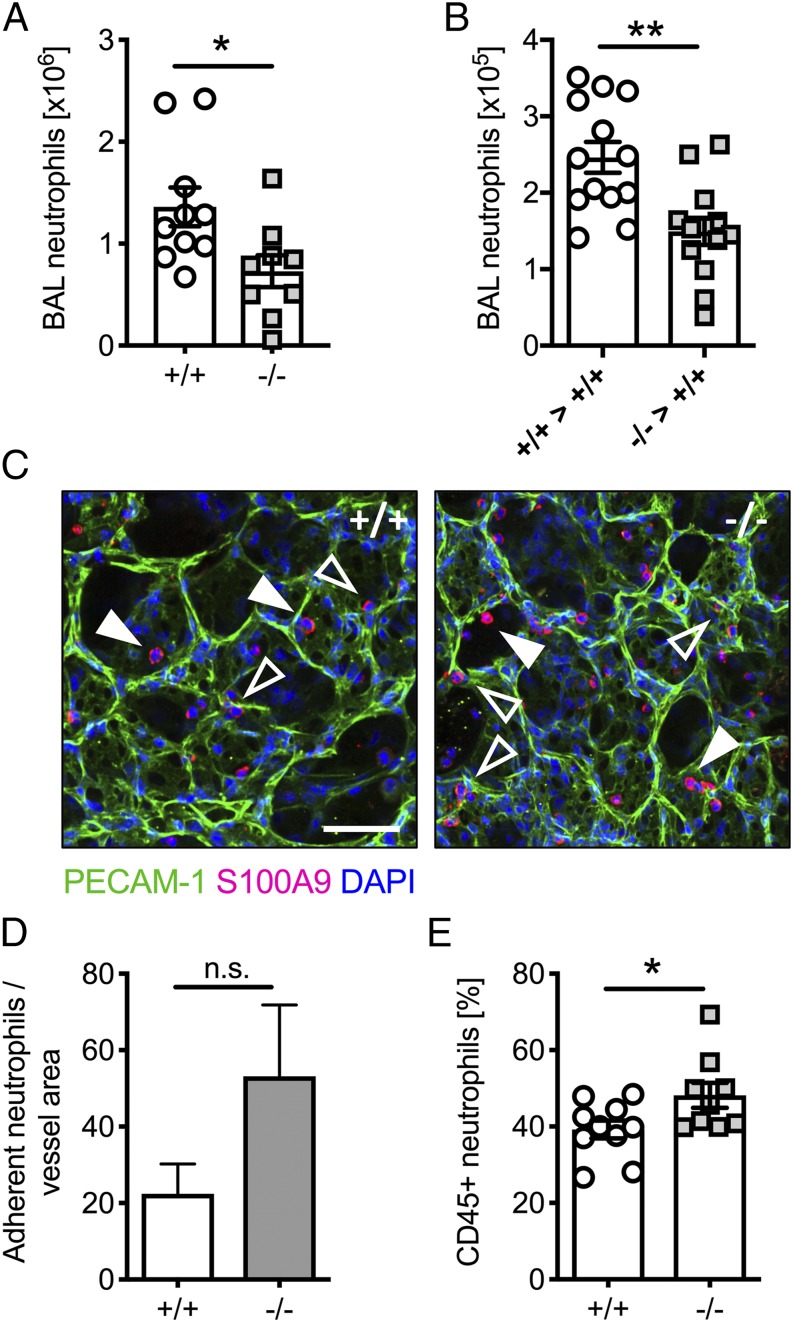
ARAP3 promotes neutrophil recruitment and transendothelial migration in a model of acute lung injury. Cre was induced by repeated tamoxifen dosing of inducible *Arap3*-knockout (^−/−^) and inducible Cre mice (^+/+^) or their bone marrow chimeras as indicated. (**A** and **B**) ALI was induced in control and ARAP3-deficient mice (A) or their bone marrow chimeras (B) by intratracheal administration of LPS. Neutrophil numbers retrieved from BAL are plotted. (**C** and **D**) Agarose-perfused, LPS-inflamed lungs were fixed and precision-sliced, and endothelium, neutrophils, and nuclei were labeled. Representative examples of rendered confocal image stacks are presented (C). Solid arrowheads represent alveolar neutrophils; unfilled arrowheads represent transendothelial/vascular firmly adherent neutrophils. Scale bar, 100 μm. (D) Images taken from two mice per genotype were analyzed, and neutrophils that were adhering to the vasculature or actively transmigrating were counted. Plotted numbers are normalized to the area of vasculature in the respective images. (**E**) Mice were i.v. administered fluorescently coupled anti-CD45 prior to lavaging of perfused lungs. Vessel-associated, CD45-labeled neutrophils in lung digests are plotted. (A, B, and E) Each symbol is representative of one mouse; graphs combine data obtained on at least two separate occasions. All bar graphs show mean ± SEM. The *p* values were calculated by unpaired two-tailed Student *t* tests. *p < 0.05, **p < 0.01.

We therefore analyzed neutrophil recruitment in response to LPS-induced ALI in control and ARAP3-deficient mice. We noted significantly reduced neutrophil numbers in BAL from ARAP3-deficient mice compared with controls (Fig. 6A). This held true with bone marrow chimeras, identifying the recruitment defect as neutrophil-autonomous (Fig. 6B).

**FIGURE 7.**
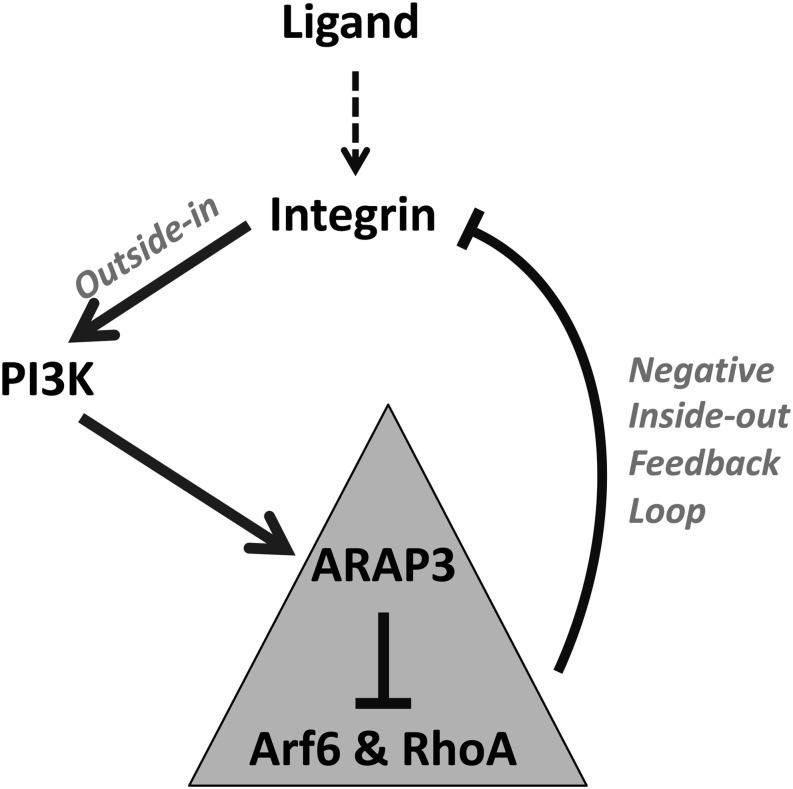
ARAP3 boosts integrin inactivation in a feedback loop downstream of PI3K in the neutrophil. Schematic depicting how integrin-mediated outside-in signaling activates PI3K to activate ARAP3, which in turn regulates integrin inactivation by negative inside-out signaling in a negative feedback loop.

To reach the alveolar space, neutrophils have to breach two barriers, the capillary wall and the alveolar epithelium. To differentiate between neutrophils that were firmly adherent to the luminal side of the vessel wall or undergoing transendothelial migration and those that were interstitial (i.e., that had extravasated but not yet breached the epithelial barrier), we generated precision slices of agarose-perfused, inflamed lung tissue, labeling endothelium and neutrophils. Microscopic analysis of such lung slices suggested that larger numbers of ARAP3-deficient neutrophils had adhered to the lung vasculature and/or were in the process of transmigrating in ARAP3-deficient lungs (Fig. 6C, 6D). We also used flow cytometry for a separate, higher-powered quantitative approach to the same question. Mice were administered a fluorescently conjugated anti-CD45 Ab i.v., labeling fully or partially intravascular leukocytes immediately prior to harvesting PBS-perfused, LPS-inflamed lungs for analysis of tissue homogenates. This identified significantly increased numbers of ARAP3-deficient neutrophils (but not macrophages) that had firmly adhered to the vessel wall or were actively transmigrating at the time of perfusion (Fig. 6E, Supplemental Fig. 3B, 3C). We concluded that ARAP3-mediated neutrophil integrin inactivation enables efficient transendothelial migration, promoting neutrophil recruitment in vivo (Fig. 7).

## Discussion

The present work identifies ARAP3 as a regulator of integrin inactivation in the neutrophil and elsewhere. Our findings place ARAP3 downstream of PI3K in a negative feedback loop that promotes integrin inactivation (Fig. 7). This mechanism enables rapid switching-off of integrins following ligand binding–induced outside-in signaling. This feedback loop operates in adherent neutrophils, in which ARAP3-dependent neutrophil activities are entirely dependent upon outside-in signaling-induced upstream PI3K activity. ARAP3 deficiency results in increased integrin activity, which in turn causes increased integrin-induced PI3K activation and downstream events.

We used integrin-dependent neutrophil chemotaxis as an experimental system in which to analyze the integrin–PI3K–ARAP3–integrin negative feedback loop in a spatiotemporal fashion. ARAP3-deficient neutrophils that chemotaxed on glass toward a point source of chemoattractant polarized PIP3 and generated pseudopods, but these were not persistently directed toward the source of chemoattractant; ARAP3-deficient neutrophils frequently displayed two (or more) poles. This is consistent with the poor integrin-dependent chemotactic migration of these cells (16). In chemotaxis on a two-dimensional matrix, class I PI3Ks are activated downstream of chemoattractant-induced GPCR signaling but also by integrin outside-in signaling. Our results suggest that ARAP3 signaling is engaged to regulate integrin inactivation in response to integrin (but not GPCR) stimulation downstream of PI3K. Our observations are consistent with the possibility that ARAP3 might simply be recruited to PIP3 in the polarized neutrophil to inactivate integrin signaling in a spatiotemporally controlled fashion, limiting further integrin-dependent localized activation of PI3K and enabling pseudopod extension. Alternatively, further players, such as PIP3 metabolizing enzymes, might also be recruited to the pseudopod to actively dephosphorylate PIP3. The functions of two PIP3 phosphatases, PTEN and SHIP1, have been analyzed in chemotaxis (29, 30, 32, 33). SHIP1 is activated and functions in adherent neutrophils, in which it regulates neutrophil spreading, chemotaxis, and PIP3 polarization, whereas PTEN is thought to regulate other features.

Physiologically, interstitial neutrophil migration is thought to be integrin-independent, whereas transendothelial migration is integrin-dependent, with some variability depending on capillary bed and stimulus (1, 2, 34). Our work suggests that in these situations, ARAP3-dependent neutrophil integrin inactivation regulates efficient neutrophil recruitment to inflammatory sites by promoting neutrophil extravasation. This identifies that neutrophil extravasation not only requires activation of integrins but, moreover, relies on their subsequent inactivation. The existence of an integrin inactivation step that regulates efficient immune responses had been predicted by an earlier report, in which rendering αLβ2 constitutively active genetically delayed T cell recruitment (9). Similarly, rendering αMβ2 constitutively active using a small molecule interfered with efficient neutrophil recruitment to inflammatory sites (8). Given that ARAP3 is highly expressed in neutrophils but not in lymphocytes (11), we speculate that integrin inactivation in lymphocytes is controlled by alternative mechanisms. ARAP1/2 are already implicated in the control of adhesion-dependent processes elsewhere (35, 36) and are expressed in lymphocytes (37), suggesting that other ARAP family member(s) might be involved in these cells.

In addition to demonstrating ARAP3-dependent inactivation of neutrophil β1 integrins, our work shows indirectly that ARAP3 also regulates neutrophil integrins that bind to substrates other than fibronectin (e.g., vitronectin, fibrinogen, and ICAM-1; data not shown and Ref. 16). ARAP3, moreover, inactivated heterologous human αIIbβ3 as well as endogenous hamster integrins in CHO cells, again in a PI3K-dependent fashion. Given that ARAP3 is expressed in CHO cells but not in platelets [which express ARAP1; (37)], αIIbβ3 is not a likely bona fide ARAP3 substrate. Rather, these observations suggest a more general function of ARAP3 downstream of PI3K in integrin inactivation. This is interesting given ARAP3’s crucial function in developmental sprouting angiogenesis and lymphangiogenesis (23, 38), processes that are not only absolutely dependent upon integrins (39) but also heavily reliant on chemotaxis, with endothelial cells migrating collectively toward VEGF. It would be interesting to test to what extent the crucial role of ARAP3 downstream of PI3K in sprouting angiogenesis is linked to integrin inactivation.

Integrin inactivation remains incompletely understood. Several scaffold proteins were shown to compete with talin for binding to integrin cytoplasmic tails in what appears to be a cell type–specific fashion. DOK-1 (40, 41) and Filamin-A (42, 43) binding to the β2 cytoplasmic tail interfered with β2 integrin activation, affecting neutrophil chemotaxis and recruitment. Similarly, SHARPIN binding to β2 in lymphocytes interfered with αLβ2 adopting high-affinity or intermediate ligand binding conformations, with its loss reducing adhesion turnover and in vitro migration and delaying homing in vivo (44). Further studies will be required to determine which, if any, of these scaffold proteins are involved in PI3K–ARAP3–mediated integrin inactivation.

## Supplementary Material

Data Supplement
