# Supplementary material for: A Negative Feedback Loop Regulates Integrin Inactivation and Promotes Neutrophil Recruitment to Inflammatory Sites
Source: J Immunol. 2019 Aug 19;203(6):1579–88. doi: 10.4049/jimmunol.1900443 (PMC6731454; doi:10.4049/jimmunol.1900443)
Supplement: Data Supplement [file JI_1900443.zip › JI_1900443_Supplemental_Figures_1.pdf]

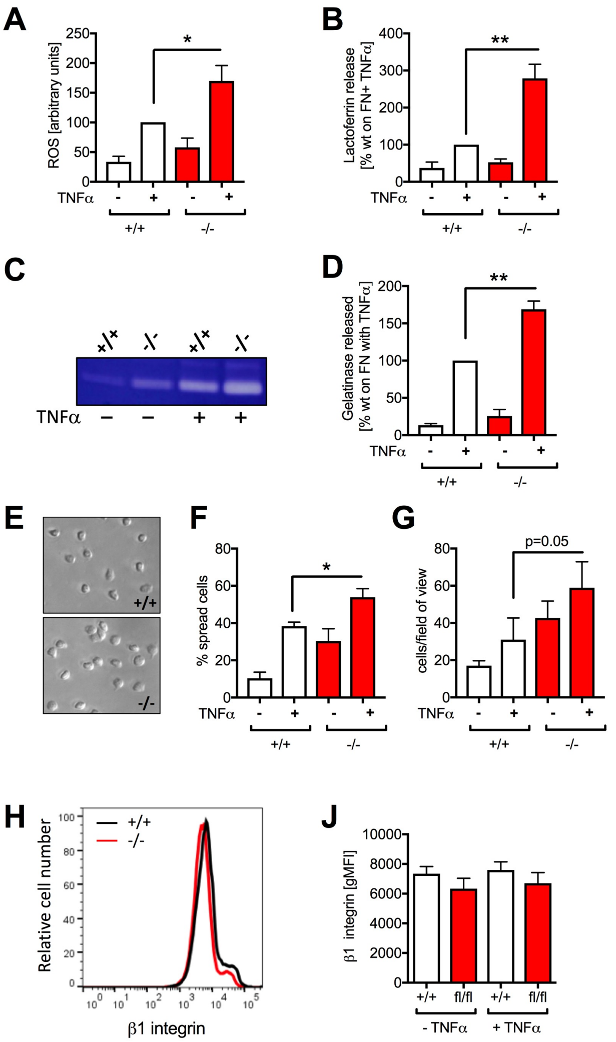

Figure S1. ARAP3-deficient neutrophils are hyperactive in integrin-dependent situations. Neutrophils were prepared from bone marrows of mock (+/+) and tamoxifen induced (-/-) inducible *Arap3* knock-out mice. (A) Analysis of ROS production of neutrophils that were stimulated by being plated onto 20 μg/ml fibronectin (FN) coated plastic in the presence or absence of 20 ng/ml TNFα in a luminol-enhanced chemoluminescence assay. This graph shows the accumulated light emission integrated from 4 separately performed experiments. (B-D) Neutrophil degranulation. Lactoferrin (B) and gelatinase (C, D) release of neutrophils that had been stimulated by being plated onto 20 μg/ml FN in the presence of absence of 20 ng/ml TNFα. A representative Coomassie-stained gel (C) and combined data from 4 separately performed experiments (B, D) are presented. Adhesion and spreading (E-G) were analyzed with neutrophils that had been plated onto 5 μg/ml FN in the presence of absence of 20 ng/ml TNFα. A representative example of adhered neutrophils is presented (E), and averaged numbers of adhered (G) and spread (F) neutrophils per field of view in the indicated conditions obtained in 3 separate experiments. (H, J) Surface expression of β1integrin was assessed by flow cytometry. A representative example plot shows cell surface β1integrin in non-stimulated neutrophils (H) and integrated data from 6 separately performed experiments with neutrophils that were or were not stimulated with 20 ng/ml TNFα (J) are plotted. All bar graphs show mean ± SEM. * P<0.05; ** P<0.01. P-values were calculated from raw data by Mann-Whitney U test.

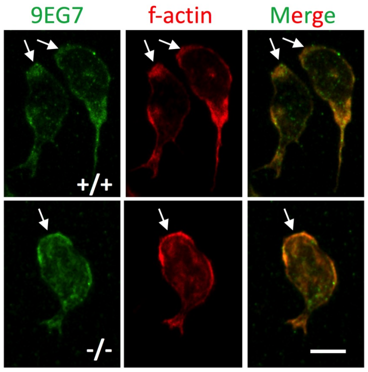

Figure S2. Activated integrins localize to the leading edge of chemoattractant stimulated neutrophils. Neutrophils were prepared from bone marrows of mock (+/+) and tamoxifen induced (-/-) inducible *Arap3* knock-out mice. Cells were allowed to settle on a glass coverslip, subjected to a bath stimulation with chemoattractant, fixed, labelled for activated β1 integrin and filamentous actin and analyzed by confocal microscopy using a Zeiss LSM780 confocal microscope and 63x objective. Representative images of control and ARAP3-deficient cells are shown; arrows indicate pseudopods. Scale bar, 5 µm.

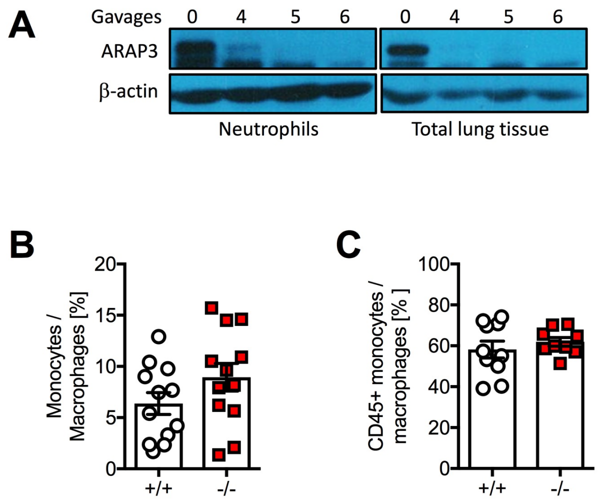

Fig S3. Deletion of ARAP3 does not affect monocyte/macrophage localization in LPS ALI. (A) Efficient long-term deletion of *Arap3* by repeat gavaging of inducible knock-outs. *Arap3*$^{fl/fl}$ *ERT2Cre*$^{+}$ mice were induced by the indicated number of gavages with tamoxifen emulsion. Mice were sacrificed 4 weeks later for analysis of ARAP3 expression (arrow) in purified BMNs and total lung tissue by Western Blot; β-actin as loading control. A representative example is presented. (B, C) Cre was induced by repeat gavaging with tamoxifen of inducible *Arap3* knock-out (-/-) or inducible Cre mice (+/+), and LPS ALI induced. Mice were intravenously administered fluorescently coupled anti-CD45 prior to lavaging of perfused lungs. Total and vessel-associated, CD45-labelled monocytes/macrophages obtained from lung digests are plotted. Each symbol represents one mouse; graphs combine data obtained from 2 separate experiments. All bar graphs show mean±SEM.
